# Nicotinamide riboside supplementation protects against maternal diabetes-associated decline in oocyte quality

**DOI:** 10.1530/REP-24-0350

**Published:** 2025-04-23

**Authors:** Chenlu Wei, Xinxin Zeng, Keer Wang, Mengchen Wang, Min Lei, Zhenye Zhu, Yining Xu, Yanqing Zhao, Qingling Yang, Yingpu Sun

**Affiliations:** ^1^ Center for Reproductive Medicine, The First Affiliated Hospital of Zhengzhou University, Zhengzhou, China; ^2^ Henan Key Laboratory of Reproduction and Genetics, The First Affiliated Hospital of Zhengzhou University, Zhengzhou, China; ^3^ Henan Provincial Obstetrical and Gynecological Diseases (Reproductive Medicine) Clinical Research Center, The First Affiliated Hospital of Zhengzhou University, Zhengzhou, China

**Keywords:** nicotinamide adenine dinucleotide (NAD+), nicotinamide riboside (NR), diabetes mellitus, infertility, oocyte, mitochondria

## Abstract

**In brief:**

NAD+ levels were reduced in streptozotocin (STZ)-induced diabetic mice, but nicotinamide riboside (NR) supplementation improved these levels in diabetic ovaries and oocytes, enhancing oocyte quality and early embryo development by improving mitochondrial function and lowering reactive oxygen species (ROS) levels.

**Abstract:**

Diabetes mellitus is strongly correlated with a decline in oocyte quality; however, noninvasive and effective methods to improve this issue have yet to be fully development. Here, we demonstrate that *in vivo* supplementation with NR 400 mg/kg/day for 14 days effectively enhances the quality of oocytes from diabetic mice induced by streptozocin 190 mg/kg by restoring nicotinamide adenine dinucleotide (NAD+) levels. NR supplementation not only improved superovulation function of diabetic mice but also improved their oocyte quality and embryonic development potential after fertilization by maintaining normal spindle structure and alleviating mitochondrial dysfunction. In addition, NR supplementation reduced ROS levels in oocytes from diabetic mice. Overall, our findings suggest that dietary NR supplementation is a viable strategy to protect oocytes from diabetes-related deterioration, thereby enhancing reproductive outcomes in maternal diabetes and improving the efficacy of assisted reproductive technology.

## Introduction

Type 1 diabetes is frequently linked to reproductive challenges in women, with up to 40% facing menstrual irregularities or reproductive issues during their lifetime (Codner *et al.* 2012, Thong *et al.* 2020, Zhang *et al.* 2024). Customarily beginning in early life, type 1 diabetes is associated with disruptions in menstrual cycles, reduced fertility and complications during pregnancy. Emerging evidence suggests that compromised oocyte quality is a major concern for women with type 1 diabetes (Kjaer *et al.* 1992, Sjoberg *et al.* 2013, Chen *et al.* 2020). In diabetic mouse models, preovulatory oocytes frequently exhibit disrupted spindle formation and misaligned chromosomes, resulting in an increased incidence of aneuploidy (Wang *et al.* 2012, Ge *et al.* 2021, Xin *et al.* 2021). In additionally, maternal diabetes disrupts cellular metabolism and mitochondrial function, resulting in increased reactive oxygen species (ROS) level and defects in meiotic progression (Wang *et al.* 2009, Jiang *et al.* 2016, Xin *et al.* 2021). Given the uncertainties surrounding the reproductive health of women with type 1 diabetes under existing management strategies, the development of a noninvasive treatment approach to address fertility issues in diabetic patients is of critical importance.

Nicotinamide adenine dinucleotide (NAD+) is a plentiful cofactor integral to various cellular metabolic processes (Yaku *et al.* 2018). Beyond its role in intermediate metabolism, it is a key regulator of vital physiological processes, such as DNA repair, stress adaptation, autophagy and maintenance of genome stability (Kennedy *et al.* 2016, Croteau *et al.* 2017). Exogenous supplementation with NAD+ precursors to elevate NAD+ level in tissues has wide positive effects on age-associated diseases and metabolic health, such as atherosclerosis, ischemic heart disease, diabetes, arrhythmogenic disorders and hypertrophic or dilated cardiomyopathies (Mills *et al.* 2016, Mitchell *et al.* 2018, Lautrup *et al.* 2019). Restoring the NAD+/NADH balance mitigated protein hyperacetylation and prevented the onset of diabetic cardiomyopathy in *Ndufs4* mice (Chiao *et al.* 2021). Notably, studies in rodents have indicated that restoring NAD+ levels may ameliorate glucose intolerance and lipid profiles and help prevent diabetes mellitus (Yoshino *et al.* 2011, Oka *et al.* 2021). Nicotinamide riboside (NR), the precursor of NAD+, synthesizes NAD+ through the salvage pathway and has been identified in dietary sources (Yang *et al.* 2020, Covarrubias *et al.* 2021). Indeed, our previous studies have demonstrated that NR supplementation can alleviate mitochondrial function and boost both the quality and quantity of oocytes by restoring age-related declines in NAD+ levels (Yang *et al.* 2021, Li *et al.* 2023). Supplementing high-fat diet mice with NR elevated NAD+ levels, which subsequently improved mitochondrial functions in oocytes through *Sirt3*-dependent pathway (Yang *et al.* 2021). Significant advances have been made in applying NAD+ in diabetes management and NR in addressing reproductive dysfunction; however, the impact of NR on female reproductive health in diabetes remains inadequately understood.

Our study identified a reduction of NAD+ levels in streptozotocin (STZ)-induced diabetic mice, while supplementation with NR increased NAD+ levels in diabetic ovaries and oocytes, thereby improving reproductive function of diabetic mice. NR supplementation improved oocytes quality and early embryo developmental potential by enhancing mitochondrial function and reducing ROS levels. Overall, our findings indicate that NR is an effective approach to mitigate diabetes-induced oocyte deterioration.

## Materials and methods

This study was approved by the Ethics Committee of the First Affiliated Hospital of Zhengzhou University. All research involving animal subjects adhered strictly to relevant ethical guidelines.

### Animal treatment

Eight-week-old female Institute of Cancer Research (ICR) mice were purchased from SKBEX Biology (China) and were managed according to the guidelines set by the Institutional Animal Care and Use Committee at the First Affiliated Hospital of Zhengzhou University. A diabetic mouse model was established by administering a single dose of streptozocin (190 mg/kg, YEASEN, China) to 8-week-old female ICR mice. Blood glucose levels were measured in tail-blood samples using an Anwen+ glucose analyzer (Sinocare Inc., China) 7 days after injection. Mice with glucose levels exceeding 16.7 mmol/L were classified as diabetic and subsequently fed a formulated grain diet with or without NR (BioChemPartner, China) supplementation at 400 mg/kg/day for 14 days, meanwhile measured blood glucose levels every 7 days to ensure the maintenance of their condition.

### NAD+ detection

According to the provided protocol, quantification of NAD+ was performed using the NAD/NADH Assay Kit (Abcam, UK). Ovarian tissues were processed in lysis buffer, and centrifugation was used to separate the supernatant. The collected samples were pre-heated with the extraction buffer at 37°C for 10 min. Subsequently, the appropriate extraction and reaction mixtures were combined with the supernatant and left to incubate for 2 hours at ambient temperature. Fluorescence measurements were conducted using a microplate reader configured with excitation at 540 nm and emission at 590 nm. NAD+ concentrations were subsequently determined by comparing the fluorescence intensity to a standard curve.

### Estrous cycle

For 14 consecutive days, vaginal smears were obtained from 8-week-old mice and processed with hematoxylin and eosin staining. The stained samples were then analyzed using an inverted microscope (Nikon, China). The stages of the estrous cycle were identified using established cytological and histological criteria (Byers *et al.* 2012). The following stages were distinguished: proestrus (presence of predominantly nucleated epithelial cells), estrus (predominance of cornified epithelial cells without nuclei), metestrus (a mix of cell types, including nucleated epithelial cells, cornified cells and leukocytes) and diestrus (dominance of leukocytes with minimal epithelial or cornified cells).

### Measurement of sex hormone levels

Blood samples were collected from the retro-orbital sinus, and the resulting serum was separated and stored at −80°C until analysis. To minimize the influence of hormonal fluctuations during the estrous cycle, and to obtain accurate and comparable hormone levels, blood samples were collected during the diestrus period of estrous cycle. The concentrations of serum anti-Müllerian hormone (AMH), luteinizing hormone (LH), follicle-stimulating hormone (FSH), estradiol (E2) and progesterone were measured using enzyme-linked immunosorbent assays (ELISA), following the manufacturer’s protocol (CUSABIO, China).

### MII oocytes collection

Superovulation was induced in female mice through an intraperitoneal injection of 7.5 IU pregnant mare serum gonadotropin (PMSG, Solarbio), followed by a subsequent injection of 7.5 IU human chorionic gonadotropin (hCG, Solarbio) administered 48 h later. Following 14–16 h, the mice were sacrificed and oviducts were extracted. Using M2 medium (Nanjing Aibei Biotechnology, China), cumulus–oocyte complexes (COCs) were obtained from the oviductal ampullae. Granulosa cells were then isolated by aspirating the ovaries with M2 medium containing 1% hyaluronidase (Solarbio, China).

### *In vitro* fertilization and embryo culture

COCs were used for *in vitro* fertilization by transferring them into a designated fertilization medium (COOK, USA). Male mice, aged 3 months, were euthanized, and their epididymides were dissected to release sperm into HTF medium (Nanjing Aibei Biotechnology, China). Following a 1-hour capacitation at 37°C under 5% CO_2_ conditions, spermatozoa were added to the fertilization medium and allowed to interact with the oocytes for 6 h. Fertilized oocytes with normal morphology were then moved to KSOM medium (Nanjing Aibei Biotechnology, China) to assess embryo formation across different developmental stages. Fertilization rate and cleavage rate were calculated based on mature MII oocytes after fertilization operations.

### Quantification of ROS

Oocytes from the different experimental groups were exposed to 5 μM MitoSOX in M2 medium (Thermo Fisher Scientific, USA) at 37°C with 5% CO_2_ for 20 min in a dark environment. Following triple washes with M2 medium, fluorescence imaging was performed using a Zeiss LSM 700 confocal microscope (Zeiss, Germany). The ImageJ software was utilized to analyze and quantify the mean fluorescence intensity of oocytes in each group.

### Mitochondria membrane potential and distribution assay

Oocytes from each group were assessed for mitochondrial membrane potential (MMP) using 10 μM JC-1 dye kit (Beyotime Biotechnology, China) in a 100 μL solution prepared as the instructions. Incubation was performed at 37°C under 5% CO_2_ for 20 min, followed by three PBS washes. Red and green fluorescence intensities were captured using a Zeiss LSM 700 confocal microscope (Zeiss, Germany), and the red-to-green ratio was used to evaluate the MMP.

To examine mitochondrial distribution in oocytes, MII oocytes were incubated in a 250 nM solution of MitoTracker Red dye (Thermo Fisher Scientific, USA) within M2 medium at 37°C and 5% CO_2_ for 30 min. Post-incubation, the oocytes underwent three washes in M2 medium, followed by imaging with a Zeiss LSM 700 confocal microscope. Fluorescence intensity was analyzed using the ImageJ software to assess mitochondrial presence.

### Statistical analysis

Results are expressed as the mean ± SEM. Shapiro–Wilk test was used to assess the normality of the data. Statistical significance was evaluated using Student’s *t*-test, chi-square test or one-way ANOVA via the SPSS (Version 22.0, SPSS Inc., USA), with *P* < 0.05 indicating significance.

## Results

### NR supplementation increased NAD+ levels in ovaries and oocytes and improved estrous cycle in diabetic mice

**Figure 1:**
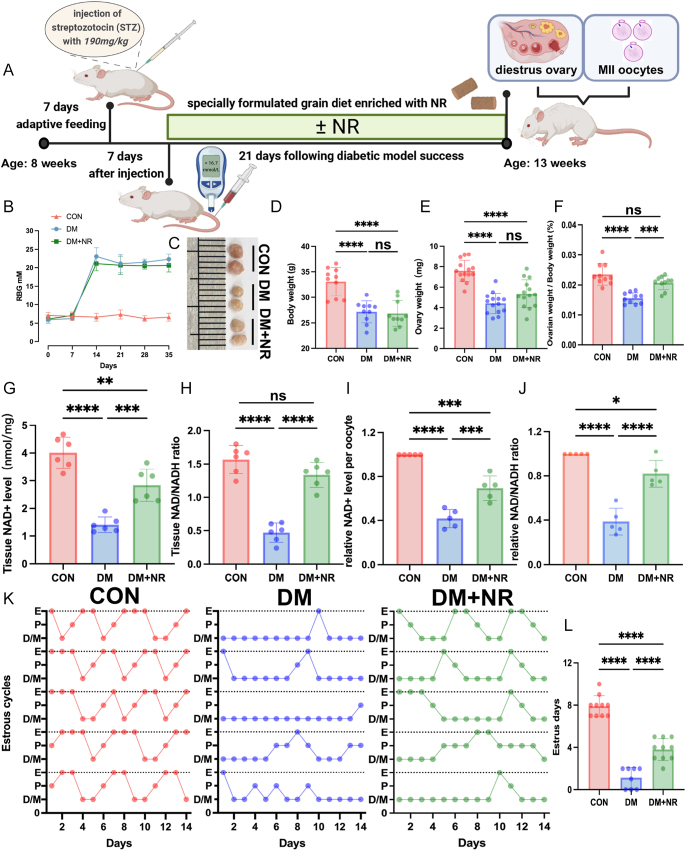
Effect of NR supplementation on NAD+ content and certain ovarian function. (A) A timeline diagram of NR supplementation to mice and hormone injection for superovulation of oocytes. (B) Random blood glucose measurements. (C) Representative macroscopic images of ovaries from the three groups of female mice. (D) Body weight in the three groups of female mice (*n* = 12). (E) Ovary weight in the three groups of female mice (*n* = 10). (F) Ovary weight-to-body weight ratio in the three groups of female mice (*n* = 10). (G) Ovarian NAD+ level in the three groups of female mice (*n* = 6). (H) Ovarian NAD+/NADH ratio in the three groups of female mice (*n* = 6). (I) Oocyte NAD+ level in the three groups of female mice. (J) Oocyte NAD+/NADH ratio in the three groups of female mice. (K) Estrous cycle of mice from the control, DM and DM+NR groups. (L) Average number of estrous days during the 14 days of observation of mice from the control, DM and DM+NR groups. Data in (B), (D, E, F, G, H, I, J), and (L) are presented as the mean percentages (mean ± SEM) from at least three independent experiments. Statistical significance is indicated as follows: *P < 0.05, **P < 0.01, ***P < 0.001, ****P < 0.0001.

Following induction of diabetes by pancreatic islet destruction, 8-week-old mice were reared with the grain diet with or without NR supplementation for 14 consecutive days (Fig. 1A). Consequently, we obtained three groups of mice for our study, respectively, a healthy control group receiving a standard grain diet, a diabetic mellitus (DM) group also receiving a standard grain diet and a DM group receiving a grain diet supplemented with NR (DM+NR). Blood glucose levels were measured every 7 days to monitor their status, ensuring accurate assessment before advancing to subsequent experimental stages (Fig. 1B). Mice in the DM and DM+NR groups exhibited notably lower body and ovarian weights than those observed in the control group (Fig. 1C, D, E). Nonetheless, NR supplementation partially alleviated the reduction in the ovarian weight-to-body weight ratio observed in diabetic mice (Fig. 1F). We quantified NAD+ and the NAD+/NADH ratio in ovarian tissues across groups. Diabetic ovaries showed significantly diminished NAD+ levels and NAD+/NADH ratios compared to control counterparts (Fig. 1G and H). However, supplementation with NR to diabetic mice significantly increased the NAD+ content in their ovaries. In diabetic mice, oocytes showed diminished NAD+ levels and a reduced NAD+/NADH ratio compared to healthy controls. Treatment with NR substantially corrected both NAD+ levels and the NAD+/NADH ratio in these diabetic oocytes (Fig. 1I and J). As a crucial indicator of ovarian function, the estrous cycle was continuously monitored for 14 days. The results revealed that diabetic mice exhibited severe disruptions in their estrous cycles compared to control mice, with almost no entry into the estrous stage (Fig. 1K and L). However, supplementation with NR partially improved the estrous cycles of diabetic mice. In additionally, we measured hormonal profiles (AMH, LH, FSH, progesterone and E2) in CON, DM and DM+NR groups. As shown in Fig. S1A, C, D (see section on Supplementary materials given at the end of the article), no significant differences in AMH, LH and E2 levels were observed across CON, DM and DM+NR groups. Compared to CON group, progesterone and LH levels in DM mice were significantly reduced (Fig. S1B and E). NR treatment did not significantly alter progesterone levels, and while there was a trend toward an increase in LH, this change was not statistically significant. These results indicate that NR supplementation in diabetic mice improves NAD+ metabolic homeostasis and the estrous cyclicity.

### Supplementation of NR reduced oocytes cytoplasmic fragmentation and improved spindle formation

**Figure 2:**
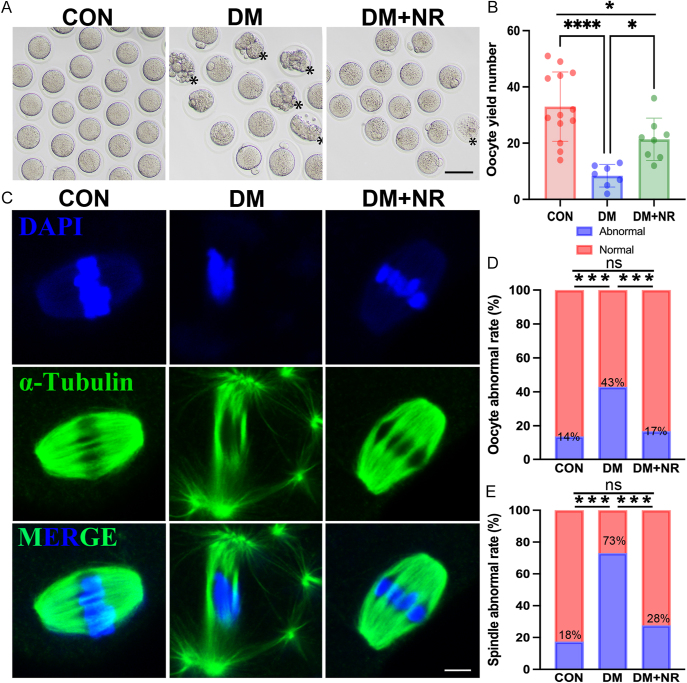
Effects of NR supplementation on the ovarian superovulatory function and oocyte quality of diabetes mellitus. (A) Representative images of MII oocytes from the three groups of female mice, with ‘*’ indicating morphologically abnormal oocytes. Scale bar, 80 μm. (B) Number of ovulated oocytes in the three groups of female mice. (C) Representative images of spindle structures. Scale bar, 5 μm. (D) Statistical graph of oocyte abnormality rate in the three groups of female mice, different letters indicate significant differences. (E) Statistical graph of spindle abnormality rate in oocytes from the three groups of female mice. Data in (B), (D) and (E) are presented as the mean percentages (mean ± SEM) from at least three independent experiments. Statistical significance is indicated as follows: *P < 0.05, **P < 0.01, ***P < 0.001, ****P < 0.0001.

We assessed the impact of NR on ovulation and oocyte quality in diabetic mice by quantifying MII oocytes and examining their morphology and spindle structures through immunofluorescence staining after superovulation. In this study, oocytes with cytoplasmic fragmentation were defined as abnormal ones. We found that diabetic mice had a significantly decreased number of ovulated oocytes and a lower oocyte maturation rate, along with an increased incidence of abnormal oocytes compared to controls (Fig. 2A and B). NR treatment effectively corrected these abnormalities, boosting both the number and structural integrity of oocytes compromised by diabetes. Moreover, a significant improvement in the number of normal MII oocytes following NR supplementation (Fig. S1F). An important criterion for evaluating oocyte quality is the structure of the spindle and chromosome alignment. We evaluated spindle integrity and chromosome positioning in MII oocytes using α-tubulin and DAPI staining after immobilization. The data indicated that 83% of control group oocytes had symmetrical spindles with well-aligned chromosomes at the equatorial plate. The rate of abnormal spindle assembly in the DM group was 73%, approximately four times higher than the rate of control group (Fig. 2C and D). Conversely, NR supplementation significantly reduced the occurrence of oocytes with abnormal spindle structures from diabetic mice. These results indicated that NR supplementation can improve ovarian superovulatory potential and reduced abnormal rate of oocytes cytoplasmic and spindle in diabetic mice.

### NR supplementation improved the fertilization ability and early embryonic development potential of oocytes in diabetic mice

**Figure 3:**
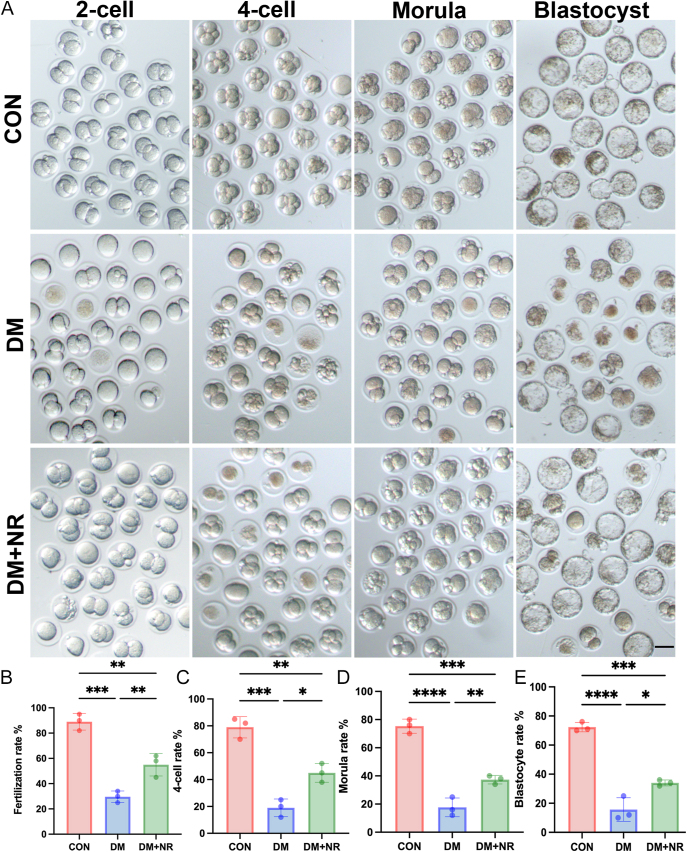
Effect of NR supplementation on the fertilization ability and embryonic development of diabetic oocytes. (A) Representative images of early embryos developed from oocytes from control, DM and DM+NR groups. Scale bar, 100 μm. (B) Fertilization rates in the control, DM and DM+NR groups. (C) Rates of four-cell embryos in the control, DM and DM+NR groups. (D) Rates of blastocyst formation in the control, DM and DM+NR groups. Data in (B, C, D) are presented as the mean percentages (mean ± SEM) from at least three independent experiments. Statistical significance is indicated as follows: *P < 0.05, **P < 0.01, ***P < 0.001, ****P < 0.0001.

After verifying the restoration of oocyte quality through NR supplementation in diabetic mice, it was also important to assess the subsequent functionality of MII oocytes as these had the most direct implications for the clinical application of assisted reproduction techniques (Holt-Kentwell *et al.* 2022). We then investigated if dietary NR supplementation in diabetic mice could improve the fertilization capacity of mature MII oocytes. *In vitro* fertilization assays showed that most control oocytes were successfully fertilized and developed into two-cell embryos, while oocytes from the DM group had significantly reduced fertilization rates compared to controls (Fig. 3A and B). As anticipated, diabetic mice treated with NR ovulated more MII oocytes with a higher fertilization rate (Fig. 3A and B). In additionally, we monitored the early embryonic development of fertilized oocytes, finding that NR supplementation significantly promoted blastocyst formation in fertilized oocytes from diabetic mice (Fig. 3A, C, D, E, F). These results demonstrated that NR improves the fertilization capability of mature oocytes from diabetic mice and enhances their subsequent embryonic development.

### NR supplementation improved mitochondrial function in oocytes from diabetic mice

**Figure 4:**
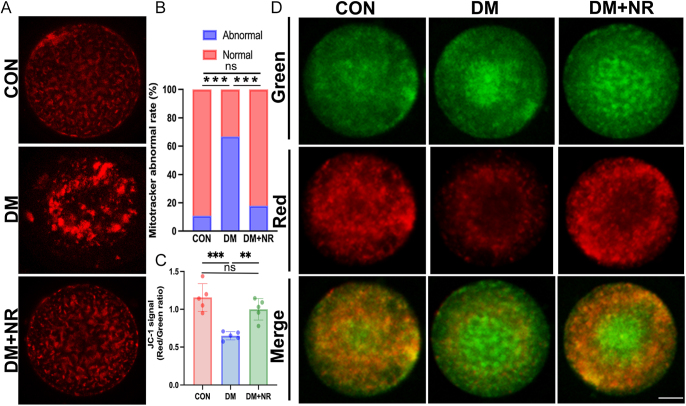
Effect of NR supplementation on mitochondrial distribution and function in diabetic oocytes. (A) Representative images of mitochondrial distribution in oocytes from control, DM and DM+NR groups. Oocytes were stained with MitoTracker red to visualize mitochondria. Scale bar, 20 μm. (B) Abnormal rates of mitochondrial distribution were recorded in oocytes from control, DM and DM+NR groups. (C) The ratio of red-to-green fluorescence intensity was calculated in oocytes from control, DM and DM+NR groups. (D) MMP (ΔΨm) was detected by JC-1 staining in oocytes from control, DM and DM+NR groups (red indicates high ΔΨm; green indicates low ΔΨm). Scale bar, 20 μm. Data in (B) and (C) are presented as the mean percentages (mean ± SEM) from at least three independent experiments. Statistical significance is indicated as follows: *P < 0.05, **P < 0.01, ***P < 0.001, ****P < 0.0001.

As the primary source of energy in oocytes, mitochondria play a crucial role, with their integrity directly affecting oocyte functionality (Yang *et al.* 2023). To explore whether improvements in oocyte quality are associated with mitochondrial condition and increased NAD+ levels, we assessed mitochondrial quality in oocytes. We firstly analyzed mitochondrial distribution using MitoTracker staining. As shown in Fig. 4A and B, mitochondria were evenly distributed throughout the cytoplasm, with some clustering near the spindle in MII stage oocytes of the control group. However, in the DM group, mitochondria exhibited abnormal aggregation and distribution. As expected, diabetic mice with NR supplementation alleviated the abnormal mitochondrial distribution in the oocytes (Fig. 4A and B).

Highly polarized mitochondria are vital for MII oocyte fertilization and for supporting early stages of embryonic development (Yang *et al.* 2021). MMP was assessed via JC-1 staining, with red fluorescence representing elevated MMP and green fluorescence indicating reduced MMP levels. As shown in Fig. 4C and D, the control group had a higher red/green fluorescence ratio, indicating optimal mitochondrial quality, whereas diabetic mice had MII oocytes with significantly reduced MMP. Conversely, MII oocytes of diabetic mice with NR supplementation restored MMP. The results suggest that NR supplementation improves post-ovulatory oocyte function in diabetic mice by reducing mitochondrial impairment.

### Supplementing diabetic mice with NR attenuated ROS levels in oocytes

**Figure 5:**
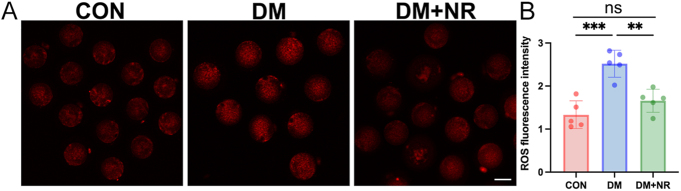
Effect of NR supplementation on ROS accumulation in diabetic oocytes. (A) Representative images of ROS levels detected by MitoSox staining in oocytes from control, DM and DM+NR groups. Scale bar, 50 μm. (B) Fluorescence intensity of ROS signals measured in oocytes from control, DM and DM+NR groups. Data in (B) is presented as the mean percentages (mean ± SEM) from at least three independent experiments. Statistical significance is indicated as follows: *P < 0.05, **P < 0.01, ***P < 0.001, ****P < 0.0001.

Mitochondrial dysfunction is widely recognized as a cause of ROS generation and oxidative stress. To compare ROS levels among different groups of oocytes, we performed MitoSox staining. Fluorescence imaging and intensity measurements revealed significantly higher ROS signals in oocytes from control compared to diabetes ones (Fig. 5A and B). In contrast, supplementary NR effectively reduced ROS accumulation in oocytes from diabetic mice.

## Discussion

Maternal diabetes, defined by consistently high blood glucose levels, is associated with compromised pregnancy outcomes and impaired embryonic development (Rodolaki *et al.* 2023). Growing evidence links diabetes to poor oocyte quality, with diabetic oocytes showing heightened oxidative stress and mitochondrial dysfunction (Gu *et al.* 2015, Li *et al.* 2022, Lu *et al.* 2022, Zhang & Wu 2023). One study of modeling by STZ injection indicated that melatonin, an effective antioxidant and free radical scavenger, helps shield oocytes from diabetes-induced autophagy and apoptosis by suppressing ROS formation (Li *et al.* 2022). Research also found that tea polyphenols lowered ROS in diabetic oocytes while boosting the expression of *Sod1*, *Sod2* and other antioxidant genes, thereby mitigating the detrimental impact of STZ-induced diabetes on oocyte quality (Lu *et al.* 2022). Although, to some extent, current clinical treatments for diabetes mellitus and advances in assisted reproductive technologies can address fertility issues, there are no noninvasive approaches available to directly improve various reproductive dysfunctions *in vivo*, such as menstrual dysfunction, ovulation disorders, poor oocyte quality and early embryonic development abnormalities of diabetes patients.

NAD+ is a vital molecule involved in cellular functions, including energy production, redox regulation, DNA repair and deacetylation of proteins (Xie *et al.* 2020). Declining NAD+ levels, commonly observed in hypertension, aging and obesity, can be counteracted by supplementation, which has been shown in preclinical models to improve health span, mitigate metabolic syndrome and decrease blood pressure (Covarrubias *et al.* 2021). Blocking NAD+ degradation via drugs or genetic methods, enhancing NAD+ levels through precursor supplementation and overexpressing NAD+-producing enzymes transgenically have been linked to significant improvements in the management of age-associated diseases and metabolic health (Abdellatif *et al.* 2021, Rotllan *et al.* 2021). Dietary NR is initially phosphorylated into nicotinamide mononucleotide (NMN) by the enzymes NRK1 and NRK2 (Sauve 2022). NMN is subsequently converted into NAD+ through the sequential actions of nicotinamide phosphoribosyl transferase and NMNAT enzymes (NMNAT1/2/3) (Alegre & Pastore 2023). One study indicated that dietary supplementation with NR effectively restores depleted NAD+ levels in the dorsal root ganglion, which helps prevent axonal degeneration associated with diabetic peripheral neuropathy (Chandrasekaran *et al.* 2022). However, this intervention does not have a significant impact on glucose tolerance, insulin concentrations or insulin sensitivity. We observed that NAD+ levels significantly drop in post-ovulatory oocytes cultured *in vitro* for 24 h; however, NR supplementation partially mitigates this decline, leading to improvements in oocyte quality and early embryo development (Li *et al.* 2023). In additionally, NMN enhances hepatic insulin sensitivity and normalizes the expression of genes associated with oxidative stress, inflammatory response and circadian rhythm, partially mediated through SIRT1 (Hong *et al.* 2020). In contrast to NR, NMN has not yet been identified in dietary sources, and its presence in serum remains controversial (Canto *et al.* 2012). This suggests that NR might be a crucial NAD+ precursor, whose levels can potentially be regulated through dietary intake. Therefore, to identify noninvasive *in vivo* treatments for improving reproductive dysfunction in diabetes patients, we opted to supplement the diet of mice with NR to explore the significance of increasing NAD+ levels.

Our study demonstrated that NAD+ levels decline in the ovaries and oocytes of diabetic mice but can be replenished through dietary NR administration. The estrous cycle involves complex rhythmic interactions between endocrine signals from the nervous and reproductive systems, coordinating ovarian hormonal and ovulatory activities (Morris *et al.* 2022). In patients with type 1 diabetes, symptoms such as oligomenorrhea and increased cycle length are common (Castellano *et al.* 2009, Thong *et al.* 2020). Research using streptozotocin-treated mice characterized by severe insulin deficiency exhibited uncontrolled hyperglycemia resulting in a catabolic state with lower serum leptin levels, which inhibit the kisspeptin expression in central nervous system, a crucial stimulator of GnRH (Castellano *et al.* 2006). Insufficient secretion of GnRH and gonadotropins has become a key factor contributing to disrupted estrous cycles and ovarian dysfunction (Brüning *et al.* 2000). As shown in our results, LH and progesterone levels were significantly reduced in the DM group compared to controls. Relatedly, one study revealed that ovaries from mice with STZ-induced diabetes were significantly smaller compared to those of control mice, accompanied by decreased expression of follicle-stimulating hormone receptor and luteinizing hormone/choriogonadotropin receptor (Lee *et al.* 2019). Although NR supplementation did not completely normalize the estrous cycle in diabetic mice, it did partially correct the severely disrupted estrous cycle, improving the condition from a state of almost complete anestrus. We hypothesize that elevated NAD+ levels in the ovary may enhance the tissue’s responsiveness to gonadotropins and improve the synergistic function of various ovarian cell types.

With the improved NAD+ content in diabetic oocytes, we aimed to determine whether NR supplementation could improve the oocyte quality. One study shows that chronic hyperglycemia of type 1 diabetes may negatively impact folliculogenesis and impair ovarian function, potentially through advanced glycation end-products (AGEs) and the action of the receptors (Escobar-Morreale *et al.* 2000, Diamanti-Kandarakis *et al.* 2007). Consistent with previous studies, our findings confirmed that diabetes mellitus severely impairs folliculogenesis, ovulation and oocytes maturation (Li *et al.* 2022, Lu *et al.* 2022). Expectedly, NR treatment increased the yield of ovulated and mature oocytes with decreasing fragmentation rate, highlighting its potential as a strategy to enhance fertility in aging females and improve oocyte maturation for assisted reproductive technology (ART) applications.

Our findings further indicated that dietary NR supplementation significantly improves spindle morphology in oocytes from mice with diabetes mellitus. To complete oocyte maturation and ovulation, as one of the key indicators for assessing oocyte quality, precise coordination between the spindle and chromosomes is essential (Cooke *et al.* 2003). Defects in spindle formation or chromosome alignment at this stage can produce aneuploid oocytes, a key factor contributing to spontaneous miscarriages and developmental abnormalities in embryos following fertilization (Battaglia *et al.* 1996). *In vitro* studies have demonstrated that certain antioxidants are effective in protecting post-ovulatory oocytes from spindle abnormalities (Rakha *et al.* 2022). Our previous study also indicated that *in vivo* supplementation with NR has been shown to improve spindle morphology in oocytes from obese mice (Li *et al.* 2023). Therefore, elevating NAD+ levels by supplementing with NAD+ precursors could serve as a promising therapeutic strategy for addressing diabetes-associated aneuploidy in oocytes.

Our results also indicate that NR supplementation improved the fertilization competence and embryonic development potential of oocytes compromised by diabetes. Maternal hyperglycemia has been shown to impair early embryonic development, particularly affecting the developmental shift from zygote to blastocyst in rodent studies (Diamond *et al.* 1989, Moley *et al.* 1998). *In vitro* studies reveal that two-cell embryos from control mice exhibit developmental delays when cultured in high glucose conditions compared to those in normal media, and this delay is further pronounced in two-cell embryos retrieved from diabetic mice, which continue to experience significant progression delays to the blastocyst stage (Diamond *et al.* 1991, Vesela *et al.* 1994). Interestingly, research showed that transplanting one-cell zygotes from diabetic mice into nondiabetic hosts resulted in a significant rise in congenital defects and growth delays in the offspring (Wyman *et al.* 2008). One study demonstrated that STZ-induced maternal hyperglycemia exerts a broad and early detrimental impact on embryonic development, specifically during the critical period between embryonic days 7.5 and 9.5 (ED.7.5–ED.9.5), which corresponds to 18–28 days of development in humans (Zhao *et al.* 2017). Exposure to maternal diabetes during oogenesis, fertilization and the early post-fertilization period can permanently alter fetal development, leading to structural defects. Furthermore, irregular mitochondrial distribution can cause imbalanced segregation during embryo cleavage, disrupting cytokinesis and causing cell lysis in blastomeres that receive a diminished number of organelles (Van Blerkom & Davis 2007, Ramalho-Santos *et al.* 2009). Mitochondrial defects in oocytes may be passed to the embryo, enhancing apoptotic rates in preimplantation embryos seen in diabetic mice (Eng *et al.* 2007). In addition to mitochondrial dysfunction, it was shown that disruption of endoplasmic reticulum redistribution processes in two-cells of diabetic mice prevented most early embryos from continuing to develop (Zhang *et al.* 2013). This explains why NR supplementation does not fully restore the developmental potential of early embryos. The mechanisms responsible for the early embryonic developmental deficits in diabetic mice are diverse, and while NR supplementation significantly improved oocyte quality and embryo developmental potential, it did not completely restore the cellular environment to a nondiabetic state. Consequently, NR supplementation may attenuate the predisposition of diabetes-associated oocytes to developmental abnormalities after fertilization and decrease the risk of metabolic disorders in the offspring via the preservation on mitochondria function.

Analysis indicated that diabetic MII oocytes showed a higher rate of abnormal mitochondrial aggregation and distribution. However, NR supplementation effectively reduced the frequency of these mitochondrial irregularities. Mitochondria are essential for energy production and maintaining redox balance in oocyte maturation. While free radical formation is a normal outcome of mitochondrial function, impaired mitochondria can lead to excessive free radical accumulation, resulting in oxidative stress (Schatten *et al.* 2014). High levels of ROS can directly harm mitochondrial membranes and DNA, aggravating mitochondrial impairment and triggering a cycle of increased ROS generation (Wang *et al.* 2009). Our research, consistent with earlier studies, revealed a notable decline in MMP in diabetic oocytes, highlighting the detrimental impact of maternal diabetes on mitochondrial performance. The maturation of oocytes requires substantial energy for various physiological activities. As oocytes mature, mitochondria gradually disperse throughout the cytoplasm, with some clustering around the spindle. This distribution likely supports the high energy requirements for spindle formation, division and the extrusion of the first polar body (Takahashi *et al.* 2016). Therefore, this improvement may contribute to the enhanced spindle morphology observed with NR supplementation.

Collectively, our study provides *in vivo* evidence that NR supplementation improves NAD+ levels, estrous dysfunction, oocytes quality and fertilization capability reduced by diabetes mellitus. Specifically, NR also suppresses ROS accumulation via restoring mitochondrial function. Our experiments did not explore the processes of *in vivo* embryo development or the maintenance of pregnancy, which are critical aspects of reproductive success. In our future studies, we will focus on the long-term effects of NR supplementation on fertility and pregnancy outcomes. Overall, this work establishes a theoretical framework supporting the use of NR to improve fertility outcomes in diabetic women and increase the effectiveness of ART procedures.

## Supplementary materials



## Declaration of interest

The authors declare that there is no conflict of interest that could be perceived as prejudicing the impartiality of the work reported.

## Funding

This study was funded by Key International (Regional) Cooperative Research Projects of China (81820108016), National Natural Science Foundation of China (31970800 and 32370917), Funding for Scientific Research and Innovation Team of The First Affiliated Hospital of Zhengzhou University (QNCXTD2023017), and the Graduate Independent Innovation Project of Zhengzhou University.

## Author contribution statement

YS and QY conceived and designed the study. CW and XZ designed the *in vitro* and *in vivo* experiment and conducted the primary animal experiments. CW and XZ established the animal models. CW, KW and ML validated the experiments related to estrous cycle. XZ and YX performed the analysis of meiotic processes and apoptosis. CW and ZZ conducted data analysis. XZ and YZ prepared the figures. CW drafted the related discussions. All authors contributed to the manuscript under the supervision of QY and YS. All authors participated in result discussions and commented on the manuscript.
