# Supplementary figures and images for: Nicotinamide riboside supplementation protects against maternal diabetes-associated decline in oocyte quality
Source: Reproduction. 2025 Apr 23;169(5):e240350. doi: 10.1530/REP-24-0350 (PMC12023624; doi:10.1530/REP-24-0350)

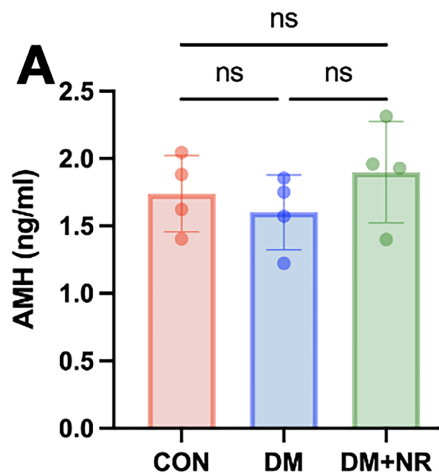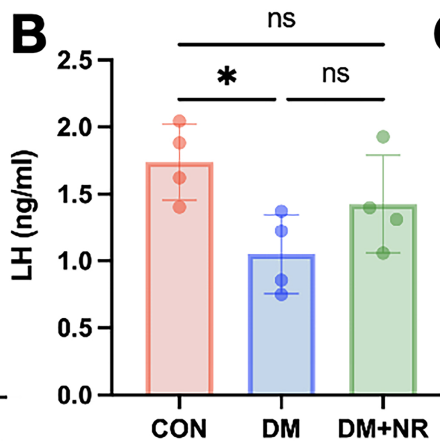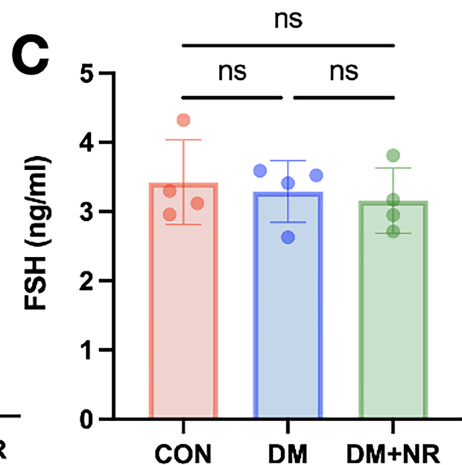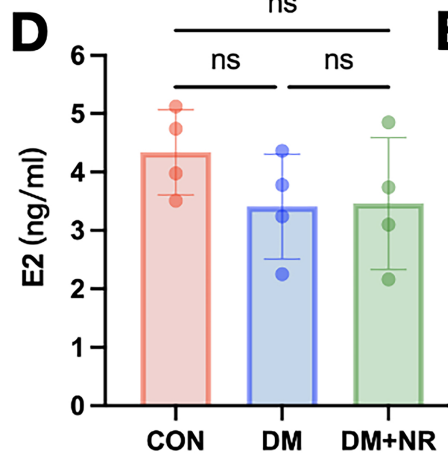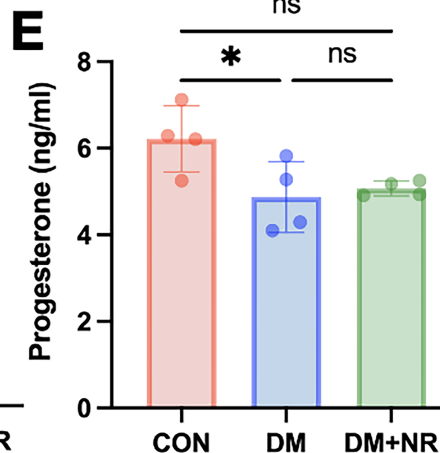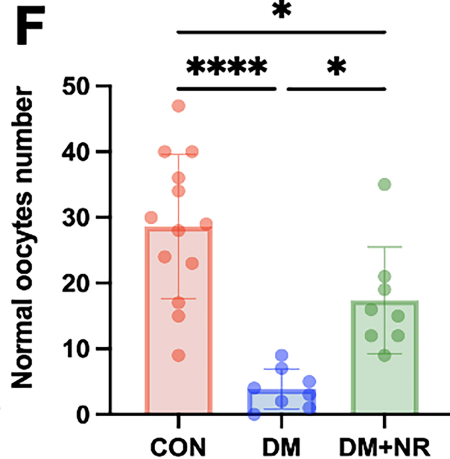

Supplement: Supplementary file 1 [file supplementary_materials.pdf]
